# P-651. The Culture Supernatant of Prevotella intermedia Caused the Ciliary Dysfunction and Delayed Clearance of S. anginosus in Vivo

**DOI:** 10.1093/ofid/ofaf695.864

**Published:** 2026-01-11

**Authors:** Koki Fukushima, Naoki Iwanaga, Takashi Kido, Ryosuke Morio, Chiaki Iketani, Ritsuko Murakami, Kazuaki Takeda, Masataka Yoshida, Shotaro Ide, Masato Tashiro, Takahiro Takazono, Kosuke Kosai, Koichi Izumikawa, Katsunori Yanagihara, Hiroshi Mukae

**Affiliations:** Nagasaki University Graduate School of Biomedical Sciences, Nagasaki, Nagasaki, Japan; Nagasaki University Hospital, Nagasaki, Nagasaki, Japan; Nagasaki University Hospital, Nagasaki, Nagasaki, Japan; Nagasaki University Graduate School of Biomedical Sciences, Nagasaki, Nagasaki, Japan; Nagasaki University Graduate School of Biomedical Sciences, Nagasaki, Nagasaki, Japan; Nagasaki University Graduate School of Biomedical Sciences, Nagasaki, Nagasaki, Japan; Nagasaki University Hospital, Nagasaki, Nagasaki, Japan; Nagasaki University Hospital, Nagasaki, Nagasaki, Japan; Nagasaki University Hospital, Nagasaki, Nagasaki, Japan; Nagasaki University Graduate School of Biomedical Sciences, Nagasaki, Nagasaki, Japan; Nagasaki University Graduate School of Biomedical Sciences, Nagasaki, Nagasaki, Japan; Nagasaki University, Nagasaki, Nagasaki, Japan; Nagasaki University, Nagasaki, Nagasaki, Japan; Nagasaki University, Nagasaki, Nagasaki, Japan; Nagasaki University, Nagasaki, Nagasaki, Japan

## Abstract

**Background:**

Several clinical studies have revealed that poor oral hygiene, contaminated with *Prevotella intermedia*, is associated with the incidence and severity of pneumonia in older adults. Using a mouse model, we previously demonstrated that oropharyngeal challenge with *Streptococcus anginosus* and the culture supernatants of *P. intermedia* (*P. int*. sup.) exacerbated pneumonia severity compared to mono-infection with *S. anginosus*. Furthermore, unbiased bulk RNA sequencing with pathway analysis revealed that *P. int*. sup.downregulated multiple mRNA expressions related to cilium movement.

**Methods:**

Female C57BL/6J mice aged 10-12 weeks were oropharyngeally inoculated of *P. int.* sup. or control medium. Mice were euthanized 36 hours post-infection, and the trachea ciliary movements, including ciliary beat frequency (CBF), ciliary beat amplitude (CBA), and ciliary beat coordination (CBC), were observed using microscopy with a high-speed camera. Also, in the same procedure, we assessed the ciliary movement under the challenge of *S. anginosus* along with *P. int.* sup or control medium. At least two blinded observers assessed these analytes, averaging five points per sample under blinded conditions. The number of bacteria in the mice's lungs was recorded 36 hours post-infection. Moreover, mice were euthanized 24 hours post-infection using the same model, and real-time RT-PCR assessed the gene expression.

**Results:**

Under conditions without *S. anginosus* infection, CBA was significantly decreased in the *P. int*. sup. group compared with the control medium group (p < 0.05). The CBC and CBF showed no significant difference. Under conditions with *S. anginosus* infection, the CBA and CBC were decreased in the *P. int*. sup. group (p < 0.05). The CBF exhibited no significant difference. The number of bacteria in the mice’s lungs was significantly increased in the *P. int*. sup. group (p < 0.05) . Genes related to ciliary function, such as *Drc3*, *Hydin*, and *Dnah11,* were significantly downregulated in the *P. int*. sup. group (p < 0.05 for each) .

**Conclusion:**

*P. int*. sup. dysregulated ciliary movement in the mice trachea, with further worsening observed under *S. anginosus* infection. Collectively, *P. int*. sup. delayed the ciliary clearance of *S. anginosus.*

**Disclosures:**

All Authors: No reported disclosures

